# Proteome and Phosphoproteome Profiling Reveal the Toxic Mechanism of *Clostridium perfringens* Epsilon Toxin in MDCK Cells

**DOI:** 10.3390/toxins16090394

**Published:** 2024-09-14

**Authors:** Nan Yue, Jing Huang, Mingxin Dong, Jiaxin Li, Shan Gao, Jing Wang, Yingshuang Wang, Dongxue Li, Xi Luo, Tingting Liu, Songyang Han, Lina Dong, Ming Chen, Jinglin Wang, Na Xu, Lin Kang, Wenwen Xin

**Affiliations:** 1 State Key Laboratory of Pathogen and Biosecurity, Institute of Microbiology and Epidemiology, AMMS, Beijing 100071, China; yn18795116327@163.com (N.Y.); ljx1658675586@163.com (J.L.); gaoshan845@163.com (S.G.); amms_wj@163.com (J.W.); dongxueli@163.com (D.L.); luoxi18907093302@163.com (X.L.); liutingtingwushi@163.com (T.L.); hansongyang0.0@outlook.com (S.H.); donglina2024@163.com (L.D.); chenming3901@163.com (M.C.); wjlwjl0801@sina.com (J.W.); kanglin@bmi.ac.cn (L.K.); 2 National Key Laboratory of Intelligent Tracking and Forecasting for Infectious Diseases, National Institute of Environmental Health, Chinese Center for Disease Control and Prevention, Beijing 100020, China; huangjing@nieh.chinacdc.cn; 3 Changchun Veterinary Research Institute, Chinese Academy of Agricultural Science, Changchun 130122, China; realdmx311@163.com; 4 Health Bureau of Nanguan District, Changchun 130022, China; wangys19@mails.jlu.edu.cn; 5 Academic Affairs Office, Jilin Medical University, Jilin 132013, China

**Keywords:** epsilon toxin, proteomics, phospho-proteomics, toxic mechanism, SRSF1, spliceosome

## Abstract

Epsilon toxin (ETX), a potential agent of biological and toxic warfare, causes the death of many ruminants and threatens human health. It is crucial to understand the toxic mechanism of such a highly lethal and rapid course toxin. In this study, we detected the effects of ETX on the proteome and phosphoproteome of MDCK cells after 10 min and 30 min. A total of 44 differentially expressed proteins (DEPs) and 588 differentially phosphorylated proteins (DPPs) were screened in the 10 min group, while 73 DEPs and 489 DPPs were screened in the 30 min group. ETX-induced proteins and phosphorylated proteins were mainly located in the nucleus, cytoplasm, and mitochondria, and their enrichment pathways were related to transcription and translation, virus infection, and intercellular junction. Meanwhile, the protein–protein interaction network screened out several hub proteins, including SRSF1/2/6/7/11, SF3B1/2, NOP14/56, ANLN, GTPBP4, THOC2, and RRP1B. Almost all of these proteins were present in the spliceosome pathway, indicating that the spliceosome pathway is involved in ETX-induced cell death. Next, we used RNAi lentiviruses and inhibitors of several key proteins to verify whether these proteins play a critical role. The results confirmed that SRSF1, SF3B2, and THOC2 were the key proteins involved in the cytotoxic effect of ETX. In addition, we found that the common upstream kinase of these key proteins was SRPK1, and a reduction in the level of SRPK1 could also reduce ETX-induced cell death. This result was consistent with the phosphorylated proteomics analysis. In summary, our study demonstrated that ETX induces phosphorylation of SRSF1, SF3B2, THOC2, and SRPK1 proteins on the spliceosome pathway, which inhibits normal splicing of mRNA and leads to cell death.

## 1. Introduction

Epsilon toxin (ε-toxin, ETX), produced by *Clostridium perfringens* types B and D [1], is the causative agent of enterotoxaemia in animals [2,3] and is also considered to be related to multiple sclerosis in humans, causing human health threats and economic losses to the global livestock industry [4,5]. Due to its high toxicity, ETX has been classified as a potential bioterrorism or bio-warfare agent by the Centers for Disease Control and Prevention of the United States. ETX has been listed as one of the potential biological warfare agents by the Centers for Disease Control and Prevention of the United States and is also listed as a category B bioterrorism threat [6]. In in vivo challenge experiments in mice, it was found that the median lethal dose of ETX to mice was 70–110 ng/kg [7,8].

ETX is composed of 311 amino acids and has a molecular weight of 32.7 kDa. At first, it is simply an inactive proETX that can be activated by extracellular serine-type enzymes [9]. Specifically, these enzymes hydrolyze to remove N-terminal and C-terminal peptides, allowing ETX to be converted to an activated form with toxic effects. Studies have shown that ETX can cause Madin Darby canine kidney (MDCK) cells to swell and rupture, eventually leading to cell death [10]. In recent years, an increasing number of cells have been found to be susceptible to ETX including the Caucasian renal leiomyoblastoma (G-402) cell line [11], the human kidney cell line ACHN [12], the murine renal cortical collecting duct principal cell line mpkCCDcl4 [13], and human erythrocytes [14]. However, MDCK cells are still the cells most used in ETX studies because they are the most sensitive [15].

The exact mechanisms of action of ETX on target cells are barely understood. Previous studies have shown that ETX can directly form pores in the target cell membrane and is a typical pore-forming toxin [10,16,17,18]. The formation of pores causes the exchange of materials between the inside and outside of the cell to be disrupted, which leads to the death of the cell [12]. However, some recent studies have found that ETX may also exert toxic effects through other pathways of action [12,14,19,20]. For example, Wioland has reported that ETX can cause oligodendrocyte demyelination [20]. Gao demonstrated that ETX can cause hemolysis in human erythrocytes by activating P2 receptors on the surface of erythrocytes [14]. Interestingly, P2 × 7 and P2Y13 inhibitors inhibited hemolysis but not ETX space formation [14]. In addition, it has been suggested that oxidation of nucleotide-sensitive ICln chloride channels contributes to ETX-induced hemolysis [21]. These studies together indicate that ETX leads to cell death mode not only through the formation of membrane pores but also through a variety of other effective pathways.

Most of the previous studies on ETX were on the pore-forming mechanism of ETX itself and on various mutants. To our knowledge, this is the first joint proteomic and phosphorylation proteomic study of the effects of ETX on MDCK cells. In this study, we profiled cell responses to ETX intoxication by performing quantitative proteomics and phospho-proteomics using MDCK cells to find clues about the pathogenesis of ETX.

## 2. Results

### 2.1. Pre-Assessment of Phosphorylation on MDCK Cells and Workflow

**Figure 1:**
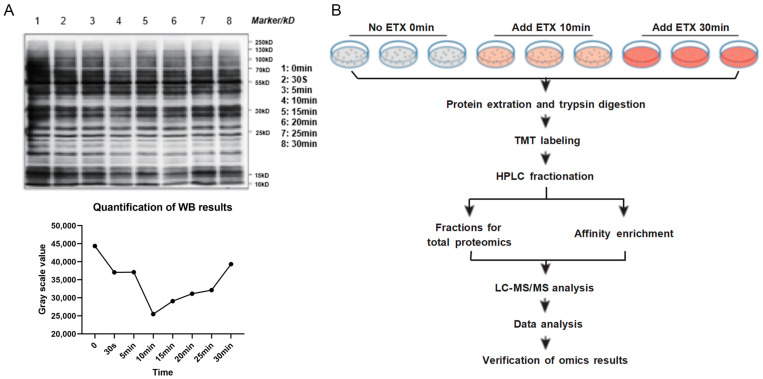
Pre-assessment of phosphorylation and workflow. (**A**) Western blotting result with pan anti-phosphotyrosine; WB results were quantified using image J software (version number Java 1.8.0–112). Three times independent gel was run for phosphoproteins. (**B**) Workflow of global proteome and phosphorylome analyses of ETX-treated MDCK cells. Three independent LC-MS/MS biology experiments were performed for each group.

To determine the point times of ETX effects on MDCK cells, immunoblotting was performed. The result is shown in Figure 1A; after being exposed to ETX for 10 min, the phosphorylated proteins were reduced. However, at 30 min, the number of phosphorylated proteins was increased. Therefore, we chose the 10 min and 30 min intervals for the following experiments. The flow chart of quantitative proteome and phosphorylated proteome is shown in Figure 1B.

### 2.2. Screening of Differentially Expressed Proteins and Differentially Phosphorylated Proteins

**Figure 2:**
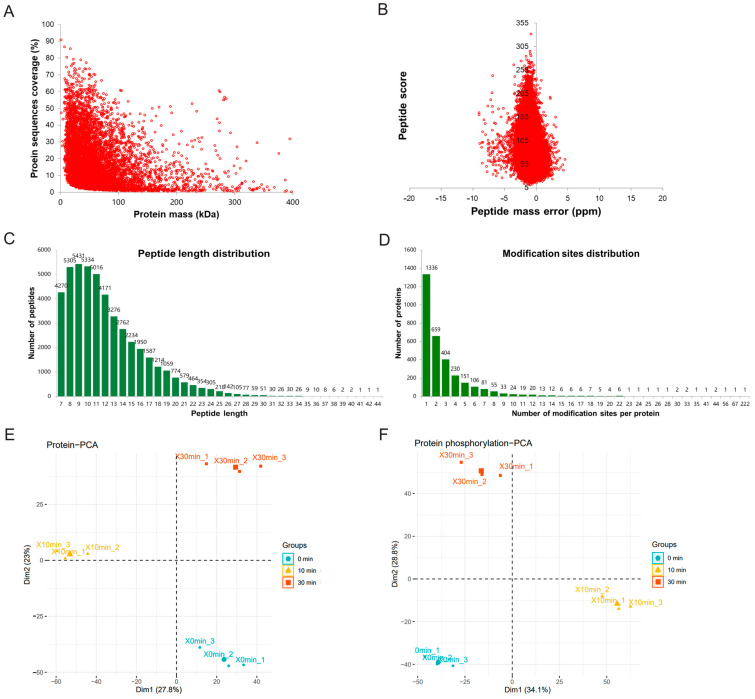
Quality control of identified proteins and phosphorylated proteins. (**A**) Relationship between protein coverage and molecular weight. (**B**) Mass accuracy distribution of mass spectrometer. (**C**) Length distribution of all identified peptides. (**D**) Distribution of phosphorylated proteins based on the number of phosphorylation sites. PCA diagram of quantitative proteomic data (**E**) and phosphorylated proteomic data (**F**).

By TMT (Tandem Mass Tags) labeling, affinity enrichment, and mass spectrometry, 6086 proteins were identified, of which 5397 proteins contained quantitative information (Table S1). We have identified 9989 phosphorylation sites, respectively, in 3199 of the proteins, of which 4916 loci with quantitative information correspond to 1688 proteins (Table S2). This indicates that the larger the protein molecular weight, the smaller the coverage (Figure 2A). First, the accuracy of the mass spectrometer was normal after measurement (Figure 2B) because most of the spectra have a first-order mass error within 10 ppm. This is also consistent with the law of tryptic hydrolysis because they are mostly present in a certain number of amino acids (about 7–20). The length of the peptide meets the requirements of mass spectrometry identification (Figure 2C). We calculated and counted the number of phosphorylation sites on proteins one by one. The protein with only one phosphorylation site accounted for 41.8% of all phosphorylated proteins. The SRRM2 protein had 222 phosphorylation sites and was the protein with the most phosphorylation sites (Figure 2D). The PCA (principal component analysis) diagrams of quantitative proteomics and quantitative phospho-proteomics show that each treatment group (MDCK cells treated with ETX for 0, 10, and 30 min) was clustered within the group and significantly separated between the groups. It indicated that the biological repeatability within each treatment group was good; meanwhile, there were obvious differences between groups, and it was valuable to mine differential proteins and phosphorylated proteins (Figure 2E,F).

**Figure 3:**
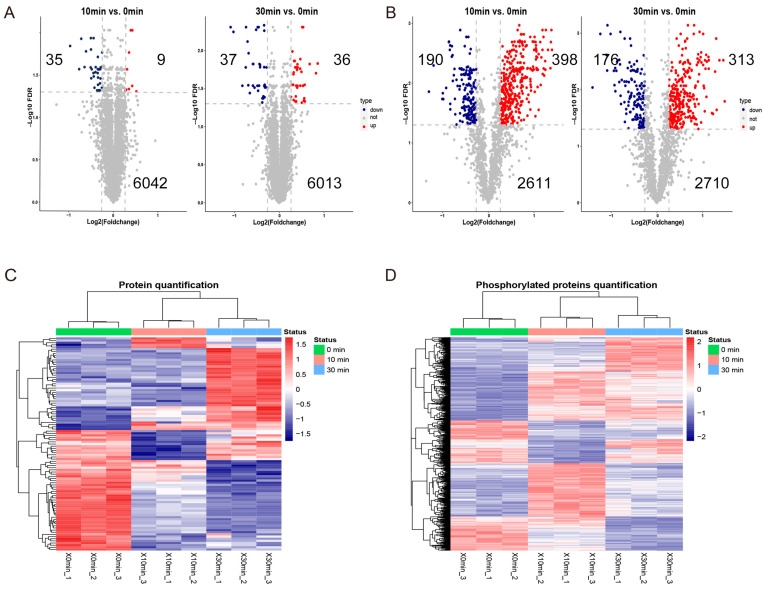
Identification of differentially expressed proteins and differentially phosphorylated proteins. (**A**) Volcano map of quantitative proteome. (**B**) Volcano map of quantitative phosphoproteome. (**C**) Heat map of three quantitative protein groups. (**D**) Heat map of three quantitative phosphorylated protein groups.

Compared with the untreated group, a total of 44 DEPs were screened out in the 10 min ETX treatment group, including 9 up-regulated proteins and 35 down-regulated proteins (Figure 3A). A total of 588 DPPs were screened out, including 398 proteins that were up-regulated and 190 proteins that were down-regulated (Figure 3B). Compared with the untreated group, a total of 73 DEPs were screened in the 30 min ETX treatment group, including 36 up-regulated proteins and 37 down-regulated proteins (Figure 3A), and a total of 489 DPPs were screened, including 313 up-regulated proteins and 176 down-regulated proteins (Figure 3B). All up- and down-regulated DPPs and DEPs are listed (Table S3). There were 17 DEPs (Table 1) and 357 differential phosphorylation sites in the intersection of the 10 min group and the 30 min group (the phosphorylation sites up- and down-regulated in the top five are shown in Table 2). From the quantitative heat map of each treatment time, it can be seen that different clustered proteins had different protein expression and modification patterns. For example, some proteins and phosphorylated proteins were continuously up-regulated with the increase of treatment time, while others were continuously down-regulated (Figure 3C,D).

### 2.3. Subcellular Localization and KEGG Enrichment Analysis

We counted the percentage of subcellular localization of DEPs and DPPs. Eighty percent of the DEPs in the 10 min and 30 min groups were localized in the nucleus, mitochondria, and cytoplasm (Figure S1A). Eighty percent of the DPPs in the two groups were located in the nucleus and cytoplasm, and the proportion of mitochondria decreased (Figure S1B).

**Figure 4:**
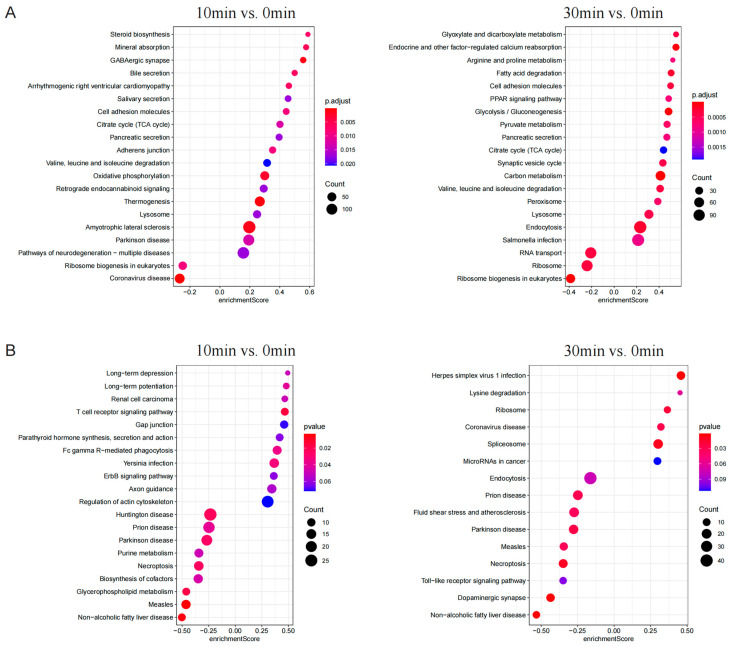
GSEA KEGG enrichment analysis. (**A**) Bubble chart of KEGG results of proteomics. (**B**) Bubble chart of KEGG results of phospho-proteomics. If more than 20 pathways are significantly enriched, the first 20 are displayed. Instead, all enrichment pathways are shown.

In order to explore the pathway involved in the toxicity of ETX, gene set enrichment analysis (GSEA) of KEGG was performed on proteomic and phosphorylated proteomic data. The five enriched pathways with the most significant up-regulated trends in the 10 min group of proteomic data were “thermogenesis”, “GABAergic synapse”, “amyotrophic lateral sclerosis”, “oxidative phosphorylation”, and “mineral absorption.” The two pathways with the most significant down-regulated trends were “coronavirus disease” and “ribosome biogenesis in eukaryotes.” The five enriched pathways with the most significant up-regulated trends in the 30 min group of proteomic data were “carbon metabolism”, “Glycolysis/Gluconeogenesis”, “endocrine and other factor-regulated calcium reabsorption”, “fatty acid degradation”, and “endocytosis”. The three pathways with the most significant down-regulated trends were “ribosome biogenesis in eukaryotes”, “ribosome”, and “RNA transport” (Figure 4A).

The five enriched pathways with the most significant up-regulated trends in the 10 min group of phosphorylated proteomic data were “T cell receptor signaling pathway”, “Yersinia infection”, “Fc gamma R-mediated phagocytosis”, “long-term potentiation”, and “renal cell carcinoma.” The five pathways with the most significant down-regulated trends were “measles”, “non-alcoholic fatty liver disease”, “glycerophospholipid metabolism”, “Huntington’s disease”, and “necroptosis.” The five enriched pathways with the most significant up-regulated trends in the 30 min group of phosphorylated proteomic data were “Herpes simplex virus 1 infection”, “spliceosome”, “ribosome”, “coronavirus disease”, and “lysine degradation.” The five pathways with the most significant down-regulated trends were “non-alcoholic fatty liver disease”, “dopaminergic synapse”, “necroptosis”, “Parkinson disease”, and “prion disease” (Figure 4B). The specific data are shown in Table S4.

### 2.4. Motif Analysis of Phosphorylation Sites

**Figure 5:**
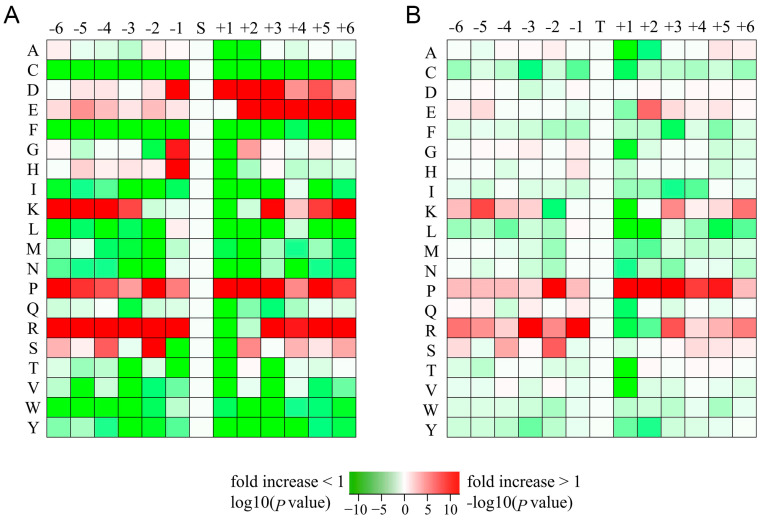
The distribution map of amino acid residues flanking the phosphorylation sites. (**A**) Distribution of amino acid residues with serine as phosphorylation site. The number of phosphorylated proteins considered was 6028. (**B**) Distribution of amino acid residues with threonine as phosphorylation site. The number of phosphorylated proteins considered was 619.

We used Motif-X to obtain the sequence characteristics around ETX-related phosphorylation sites and calculated the frequency of amino acid residues on both sides of typical phosphorylation sites (Ser/Thr sites). Taking threonine as the phosphorylation site, the frequency of Asp, Glu, Lys, Pro, and Arg in the surrounding amino acid residues was high, and the motif of Pro and Asp was highly enriched at the position of + 1 (Figure 5A, Table S5). With serine as the phosphorylation site, the frequency of Lys, Pro, and Arg in the surrounding amino acid residues was high. At the same time, the motif of Pro was highly enriched at the position of + 1 (Figure 4B, Table S5).

### 2.5. Integration Analysis of Differentially Expressed Proteins and Differentially Phosphorylated Proteins

In order to more comprehensively identify the toxic mechanism of ETX, we integrated the DEPs and DPPs of the 10 min group and the 30 min group into GO, KEGG enrichment analysis, and PPI network construction.

**Figure 6:**
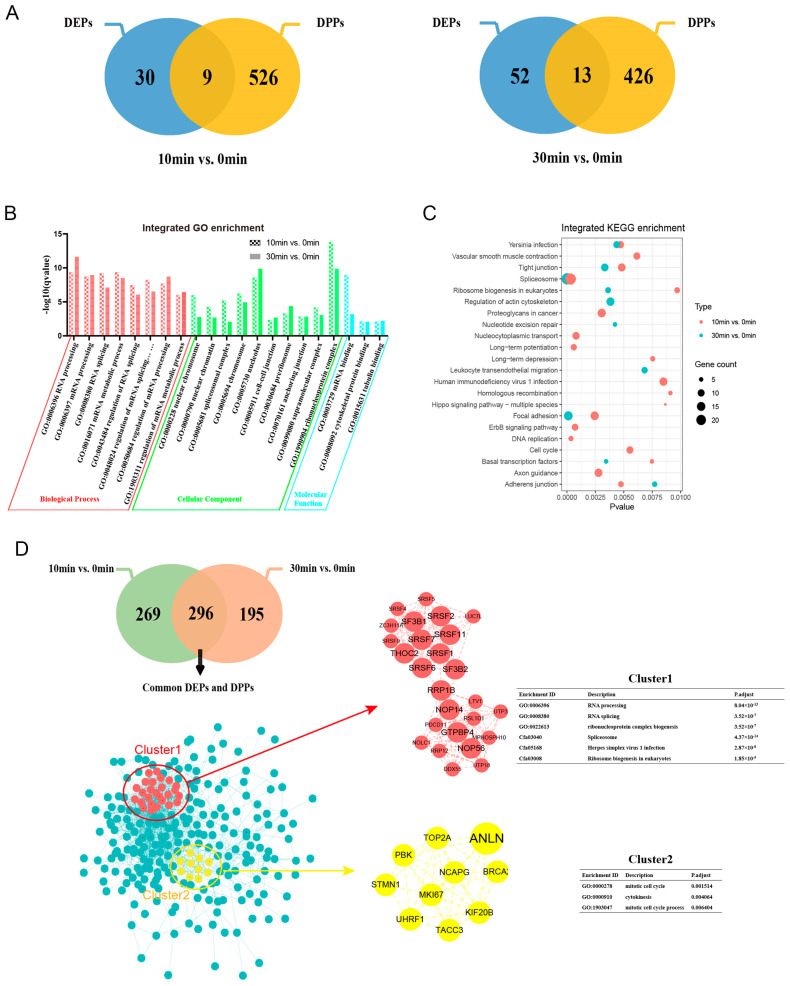
Integration analyses of differentially expressed proteins (DEPs) and differentially phosphorylated proteins (DPPs). (**A**) The union of DEPs and DPPs in the two groups. (**B**) Go terms enriched by the two groups. (**C**) KEGG pathways enriched by the two groups. (**D**) PPI network of DEPS/DPPS between the two groups. The big circle in the sub-network represents the top 20 degrees proteins in the total network. DEPs: differentially expressed proteins; DPPs: differentially phosphorylated proteins.

There were 565 DEPs and DPPs in the 10 min group and 491 DEPs and DPPs in the 30 min group (Figure 6A). The two groups of DEPs and DPPs were enriched by GO and KEGG, respectively, and the co-enriched GO pathways were visualized. The most important biological process (BP) in the two groups of cells is “RNA processing”, the most important cellular component (CC) is “ribonucleoprotein complex”, and the most important molecular function (MF) is “mRNA binding” (Figure 6B). The top three co-significant pathways were “spliceosome”, “focal adhesion”, and “tight junction” (Figure 6C).

There were 296 DEPs and DPPs in the intersection of the 10 min group and the 30 min group. The PPI (protein–protein interaction) network of intersection proteins was constructed, and the extent of each protein in the network was calculated using Cytoscape (version 3.8.2) software’s Cytotumor plug-in to screen the top 20 hub proteins (Table S6). Using the MCODE plug-in, two sub-network clusters were selected from the complex network. Cluster1 had 26 points and 140 edges and contained the 12 proteins of the top 20 hub proteins ranked by degree in the total networks; its main biological processes are RNA processing and splicesome. Cluster2 had 10 points and 36 edges, including 1 protein (ANLN) of the top 20 proteins ranked by degree in the total network. The biological process it mainly participates in is related to the cell cycle (Figure 6D).

### 2.6. Inhibitors Reduced Toxicity of ETX on MDCK Cells

After analyzing the 13 homologous key proteins of the KEGG pathway one by one in the cluster, 9 proteins could be found corresponding to the pathway (Table 3). To demonstrate whether these proteins are key to determining that ETX induced the death of MDCK cells, we conducted experiments to verify it.

**Figure 7:**
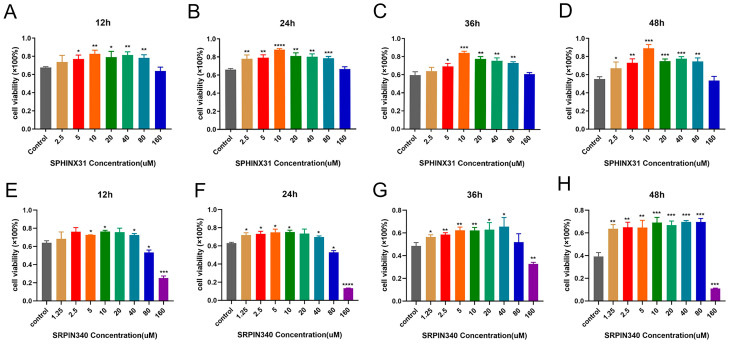
Inhibiting the expression of SRSF1 and SRPK1 can effectively inhibit the lethality of ETX on MDCK cells. (**A**–**D**) MDCK cells were treated with different concentrations of SPHINX31 for 12, 24, 36, and 48 h, and the inhibitor can improve the survival rate of ETX after attacking MDCK cells. (**E**–**H**) MDCK cells were treated with different concentrations of SRPIN340 for 12, 24, 36, and 48 h, which also improves survival after ETX attacks MDCK cells. Values are the mean ± SD (*n* = 3). At least three independent biological replicates were performed for each experiment. * *p* < 0.05, ** *p* < 0.01, *** *p* < 0.001, and **** *p* < 0.0001; this was compared to controls in each group that did not use lentivirus.

We found that these nine proteins were involved in three pathways, most of which were concentrated in the SR protein complex of the spliceosome pathway. Therefore, we selected SRPK1, a common upstream protein of this pathway, for verification. We used an inhibitor, SPHINX31, that has been shown to be effective in inhibiting the phosphorylation of serine/arginine-rich splicing factor 1 (SRSF1) and is also a selective inhibitor of serine/arginine-rich protein kinase 1 (SRPK1) [22,23]. MDCK cells were treated with different concentrations of SPHINX31 for 12, 24, 36, and 48 h before being exposed to ETX (8.93 nM), and it was found that the survival rate of MDCK cells was enhanced in a concentration-dependent manner (Figure 7A–D). In addition, SRPIN340 is also an ATP-competing SRPK inhibitor and has been shown to effectively inhibit SRPK1 expression. Similarly, different concentrations of SRPIN340 were applied to MDCK cells, and it can be seen that SRPIN340 could effectively inhibit the death of MDCK cells at low concentrations but had certain cytotoxicity when the concentration was greater than 80 μM (Figure 7E–H). The results suggest that SRSF1 and SRPK1 are involved in ETX-induced toxicity on MDCK cells.

### 2.7. SRSF1 and SRPK1 Inhibit the Toxicity of ETX to MDCK Cells

**Figure 8:**
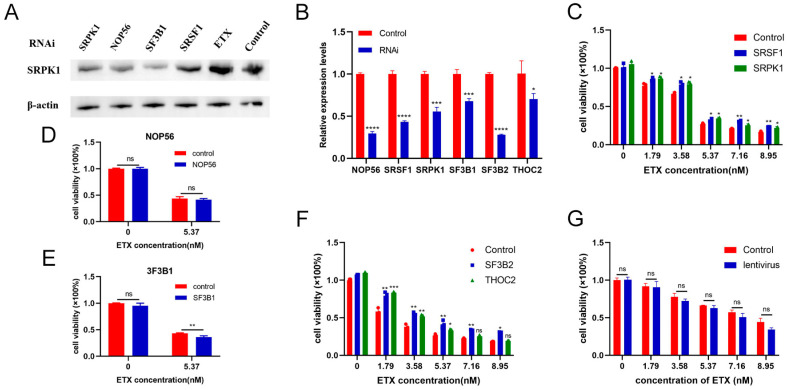
Lentiviruses using SRSF1 and SRPK1 can effectively inhibit the toxicity of ETX to MDCK cells. (**A**) ETX can increase the expression of SRPK1 protein in MDCK cells, and the four lentiviruses we synthesized can reduce the expression of SRPK1. (**B**) The relative quantitative results of RT-qPCR showed that the six lentiviruses could significantly reduce the expression levels of corresponding proteins in MDCK cells. (**C**) Lentivirus-decreased expressions of SRSF1 and SRPK1 could effectively inhibit the death of MDCK cells. (**D**,**E**) NOP56 does not play a role in cell death, and decreased SF3B1 expression can promote the death of MDCK cells. (**F**) Knockdown of SF3B2 and THOC2 could reduce the death rate of MDCK cells caused by ETX to a certain extent. (**G**) The lentiviral vector did not affect the survival of the cells. The above experimental cells were all exposed to ETX for 1 h. Values are the mean ± SD (*n* = 3). At least three independent biological replicates were performed for each experiment. * *p* < 0.05, ** *p* < 0.01, *** *p* < 0.001, and **** *p* < 0.0001; this was compared to controls in each group that did not use lentivirus.

In addition, to further identify the central proteins of MDCK that caused death by ETX, we selected six key proteins on the three key pathways obtained, NOP56, SF3B1, SF3B2, THOC2, SRSF1 and SRPK1, and designed lentivirus to knock down the expression levels of these proteins in MDCK cells, respectively (information on customized lentiviruses is provided in Table S7). The results expressed that ETX could lead to the increase of SRPK1 in MDCK cells, and the use of the four lentiviruses could reduce the expression of SRPK1 in MDCK cells (Figure 8A). In addition, the levels of these six key proteins in MDCK cells were also knocked down by corresponding lentiviruses (Figure 8B). The results of the cell cytotoxicity assay showed that the specific knockdown of SRSF1 and SRPK1 by lentivirus could effectively inhibit the cell death of MDCK cells (Figure 8C), which was consistent with the results of previous inhibitors. However, NOP56 does not play a role in cell death, and decreased SF3B1 expression can promote the death of MDCK cells (Figure 8D,E).

Additionally, to further confirm our conclusion, we selected two key proteins in the spliceosome pathway, SF3B2 and THOC2, and constructed lentiviruses to knock down their expression levels in MDCK cells. The results showed that SF3B2 could inhibit the toxicity of ETX to MDCK cells, while THOC2 could inhibit cell death at ETX = 1.79 nM, but the inhibitory effect was not significant at high concentrations of ETX (Figure 8F). The negative control of the lentivirus vector used had no effect on cell viability (Figure 8G). These results support our findings in the inhibitor experiments above that SRSF1, SRPK1, and their spliceosome pathways play critical roles in ETX-induced cytotoxicity in MDCK cells (Figure 7).

## 3. Discussion

ETX is highly toxic and can cause disease and even death in a variety of animals [24]. Because of this, it is considered a potential agent of biological and toxin warfare. After ETX poisoning, the course of the disease develops rapidly, and the mortality rate is high [25]. Therefore, it is necessary to reveal the pathogenesis of ETX, search for key signaling pathways and molecules, and strive to provide help and therapeutic targets for subsequent therapeutic research.

In the enriched ETX-related pathways, there were many transcription- and translation-related words, such as “RNA transport”, “ribosome”, “splicesome”, “RNA processing”, “mRNA metabolic process”, etc., and most of the DEPs and DPPs were located in the nucleus, cytoplasm, and mitochondria. At the same time, the core sub-network in the PPI network was also enriched in “RNA processing”, “RNA spreading”, and other processes, indicating that the toxicity of ETX may be closely related to interfering with the transcription and translation of host cells. In order to verify the role of these hub proteins in ETX poisoning, we systematically summarized the pathways where these proteins were located, and they were found to be almost concentrated in three pathways: Herpes simplex virus 1 infection and spliceosome and ribosome biogenesis in eukaryotes. What is more interesting is that six of these proteins (SF3B1/2, SRSF1/2/6, and THOC2) exist in the “spliceosome” pathway. The spliceosome, which removes introns from genes and connects exons together to synthesize pre-mRNA, plays a crucial role in gene expression [26,27].

Abnormal RNA splicing causes a variety of diseases [28]. Interestingly, SF3B1/2 and SRSF1/2/6 are both present in the SR protein complex of this pathway; SR proteins promote the recruitment of spliceosome components through protein–protein interactions to promote early spliceosome assembly, and these functions are regulated by reversible phosphorylation [29]. Therefore, we suspect that these proteins may be the key proteins that determine ETX poisoning. Studies have proved that high expression of splicing factor SRSF1 can promote the occurrence and development of lung cancer [30], breast cancer [31], pancreatitis [32], and other diseases and cancers, indicating that SRSF1 is a key protein in the regulation of cancer cell expression. Studies have shown that the abnormal expression of the SRSF2 gene is strongly correlated with acute myeloid leukemia (AML) [33], liver cancer [22,34], lung cancer [35], and other diseases. Recent studies have found that HSV-1 (herpes simplex virus-1) infection can up-regulate SRSF2 levels, and SRSF2 can negatively regulate HSV-1 transcription [36]. Therefore, SRSF2 is expected to be a therapeutic target for a variety of diseases. Multiple alternative splicing of SRSF6 also provides therapeutic targets for diseases such as rectal cancer [37], gastric cancer [38], and alcoholic liver disease [39]. SF3B1 is an important component of the spliceosome. Reduced expression of this gene can reduce the incidence of many human cancers and can also slow the progression of cancer [40,41,42]. But we have only found a finished inhibitor of the SRSF1 protein, SPHINX31, which can inhibit the phosphorylation of SRSF1, and no specific antibodies against them have been found.

We then went further up the pathway and found that the common upstream protein of these proteins was SRPK1. SRPK1 is a protein that can be involved in mRNA processing, including, of course, the alternative splicing mentioned earlier [43]. One of the unique features of SRPK1 is that it phosphorylates proteins containing the SR domain. Studies have shown that the high expression of SRPK1/2 is the cause of enhanced phosphorylation and nuclear translocation of SRSF1 [44]. Therefore, we have reason to believe that SRPK1 plays a key role in mediating ETX-induced MDCK cell poisoning. Interestingly, we found that SRPIN340 is an ATP-competitive SRPK inhibitor that inhibits SRPK1 activity. Therefore, in our results, we found that the reduction of SRSF1 and SRPK1 activity can effectively inhibit the death of MDCK cells induced by ETX. Lentivirus knockdowns of the two proteins produced the same results, and the increase in cell survival was more pronounced in the high-concentration ETX group.

Based on the above results, we suspect that when ETX acts on MDCK cells, SRPK1-specific kinase phosphorylates multiple proteins in the SR protein complex, thereby inhibiting the normal splicing of mRNA in the cells and preventing the synthesis of correct proteins, resulting in cell death. As has been reported in many studies, our study also demonstrates that SRPK1 does affect the normal life cycle of cells. However, it plays an important part in cancer treatment and diagnosis [45], whether it helps in the treatment of diseases caused by ETX is unknown, and this is a question that we must continue to address. This also suggests that ETX-induced disease and injury are not as simple as the formation of pores in the membrane but have more complex damage mechanisms, and our findings are an important starting point.

Unfortunately, of the 13 proteins we screened, most currently have no finished inhibitors and antibodies, and several proteins are located in unknown pathways, making our research and validation extremely difficult. Further exploration of these unknown proteins is essential. The research on the pathogenesis of ETX needs to be ongoing. The above differential proteins can be used as potential therapeutic sites for ETX intoxication.

## 4. Conclusions

Taken together, we used quantitative proteomics and quantitative phospho-proteomics techniques to screen ETX pathogenic-related proteins, phosphorylated proteins, and some important pathways in the MDCK cell line. We identified the key proteins SRPK1, SF3B2, THOC2, and SRSF1 in ETX-induced cell death and verified that their high expression can lead to abnormal splicing of MDCK cells, leading to cell death. To our knowledge, this is the first joint proteomic and phosphorylation proteomic study of ETX on MDCK cells, which provides data support for clarifying the pathogenic mechanism, screening toxic biomarkers, and looking for therapeutic targets of ETX intoxication.

## 5. Materials and Methods

### 5.1. Recombinant Toxin and Reagents

SRPK1 Polyclonal Antibody was purchased from Immunoway (Plano, TX, USA). Horseradish peroxidase (HRP)-conjugated goat anti-rabbit IgG (H + L) antibody was purchased from Solarbio (Beijing, China). SPHINX31 (HY117661) and SRPIN340 (HY13949) were purchased from Med Chem Express Corporation (MCE, Romulus, MI, USA). SRSF1, SRPK1, SF3B1, and NOP56 four RNAi-custom lentiviruses were built by GENE Corporation (Shanghai, China). RIPA Lysis Buffer abs929 (strong) was purchased from Absin (Shanghai, China).

Recombinant toxins, GST-labeled ETX, were expressed and purified according to the basic procedures previously performed in the laboratory [14,46].

### 5.2. Cell Culture

MDCK cells were cultured in DMEM complete medium (Dulbecco’s Modified Eagle Medium, Gibco, Carlsbad, CA, USA), and the conditions of the incubator were set at 37 °C with 5% CO_2_.

### 5.3. Western Blotting

To assess the effects of ETX (0.9 nM) on MDCK cells, 1 × 10^5^/mL cells were plated in 150 mm plates for 24 h. Subsequently, the cells were incubated with ETX (0.9 nM) in DMEM for different durations (0, 0.5, 5, 10, 15, 20, 25, and 30 min) at 37 °C. After incubation, cells were washed three times with PBS. According to the manufacturer’s instructions (absin, Shanghai, China, abs9229), 25 μL of PMSF at a concentration of 100mM was added to 225 μL of RIPA lysate to make the final concentration of PMSF 1 mM. After mixing evenly, 250 μL of the mixture was added to each well, and the cells were lysed for 1 min. After which the cells attached to the bottom of the plate were scraped off with a scraper and collected into a centrifuge tube; this procedure was performed on ice. The lysates were then centrifuged at 14,000× *g* for 5 min at 4 °C. The supernatants obtained by centrifugation were collected for subsequent experiments. Samples were mixed with loading buffer and added to a 4–20% polyacrylamide SDS-PAGE gel for electrophoresis, after which they were transferred to PVDF membranes. The membranes were blocked with 5% skim milk powder for approximately 3 h at room temperature by gentle shaking and washed three times using PBST. Then, the membranes were incubated with anti-SRPK1 polyclonal overnight at 4 °C and washed three times using PBST. The membranes were incubated with goat anti-rabbit IgG secondary antibody for 1 h. Finally, the color development solution was added, and the results were analyzed and imaged using an AE-1000 cool CCD image analyzer.

The Western blotting results were quantified using image J software (version number: Java 1.8.0-112).

### 5.4. Protein Extraction

MDCK cells treated with ETX for 10 min and 30 min were collected, then lysate (8 M urea, 1% protease inhibitor) was added and crushed using ultrasonic treatment on ice for 10 s. The crushed liquid was centrifuged at 4 °C, 14,000× *g*, for 10 min. The supernatant was collected, and the protein concentration was determined.

### 5.5. Trypsin Digestion

The sample pretreatment before digestion was as follows [47]: The protein was reduced with 5 mM dithiothreitol for 30 min and then alkylated with 11 mM iodoacetamide under dark conditions for 15 min. Finally, the urea concentration of the sample was diluted to less than 2 M by adding 100 mM NH_4_HCO_3_. Then, the protein solution was digested twice, first at a 1:50 ratio of trypsin to protein overnight, followed by a 1:100 ratio of trypsin to protein for 4 h. Finally, the peptides were desalted by Strata X SPE column.

### 5.6. TMT Labeling

Tryptic peptides were first dissolved in 0.5 M TEAB. Each channel of peptide was labeled with their respective TMT reagent (based on manufacturer’s protocol, Thermo Scientific, Rockford, IL, USA, cat.90406) and incubated for 2 h at room temperature. Five microliters of each sample were pooled, desalted, and analyzed by MS to check labeling efficiency. After labeling efficiency check, samples were quenched by adding 5% hydroxylamine. The pooled samples were then desalted with Strata X SPE column (Phenomenex) and dried by vacuum centrifugation.

### 5.7. HPLC Fractionation

Separation was performed using a high pH reversed-phase HPLC on Agilent 300Extend C18 column (5 μm particles, 4.6 mm ID, 250 mm long). The 8–32% acetonitrile gradient of PH9.0 separated 60 fractions. The resulting fractions were recombined and dried, resulting in 18.

### 5.8. LC-MS/MS Analysis

The tryptic peptides were dissolved in solvent A (0.1% formic acid, 2% acetonitrile/in water), directly loaded onto a homemade reversed-phase analytical column (25 cm length, 75/100 μm i.d.). Peptides were separated with a gradient from 8% to 25% solvent B (0.1% formic acid in 90% acetonitrile) over 40 min, 25% to 35% in 14 min, and climbing to 80% in 3 min, then holding at 80% for the last 3 min, all at a constant flowrate of 700 nL/min on an EASY-nLC 1000 UPLC system (Thermo Fisher Scientific, Waltham, MA, USA).

The separated peptides were analyzed in Q ExactiveTM (Thermo Fisher Scientific, Waltham, MA, USA) with a nano-electrospray ion source. The electrospray voltage applied was 2.1 kV. The full MS scan resolution was set to 70,000 for a scan range of 350–1800 *m*/*z*. Up to 10 most abundant precursors were then selected for further MS/MS analyses with 15 s dynamic exclusion. The HCD fragmentation was performed at a normalized collision energy (NCE) of 28%. The fragments were detected in the Orbitrap at a resolution of 35,000. Fixed first mass was set as 100 *m*/*z*. Automatic gain control (AGC) target was set at 5E4, with an intensity threshold of 5E3 and a maximum injection time of 200 ms.

### 5.9. Database Search

The resulting MS/MS data were processed using MaxQuant search engine (v.1.5.2.8). Tandem mass spectra were searched against the canis SwissProt database (25,492 entries) concatenated with reverse decoy database. Trypsin/P was specified as cleavage enzyme allowing up to 2 missing cleavages. The mass tolerance for precursor ions was set as 20 ppm in First search and 5 ppm in Main search, and the mass tolerance for fragment ions was set as 0.02 Da. Carbamidomethyl on Cys was specified as fixed modification. Acetylation on protein N-terminal, oxidation on Met, and deamidation (NQ) were specified as variable modifications. TMT-10plex quantification was performed. FDR was adjusted to <1% and minimum score for peptides was set to >40.

### 5.10. Bio-Material-Based PTM Enrichment (For Phosphorylation)

Enrichment was performed using IMAC kit (ThermoFisher Scientific, Rockford, IL, USA, Cat. A32992) as follows. Peptide mixtures were first incubated with IMAC microspheres suspension with vibration in loading buffer (50% acetonitrile/0.5% acetic acid). To remove the non-specifically adsorbed peptides, the IMAC microspheres were washed with 50% acetonitrile/0.5% acetic acid and 30% acetonitrile/0.1% trifluoroacetic acid, sequentially. To elute the enriched phosphopeptides, the elution buffer containing 10% NH_4_OH was added, and the enriched phosphopeptides were eluted with vibration. The supernatant containing phosphopeptides was collected and lyophilized for LC-MS/MS analysis.

### 5.11. Standardization of Data

First, the anti-library and the contaminated library were removed. Then, the data were first laterally homogenized (replacing 0 values with NaN and dividing the intensity values of each protein in all samples by the mean of the intensity values of all proteins in all samples) and then longitudinally normalized (dividing each column by the median of that column). Finally, unique peptides were grouped (peptide segments belonging to the same protein are grouped together), and the median of quantitative values in each group of peptides was used as the quantitative value of the protein.

### 5.12. Bioinformatics Analysis

We performed principal component analysis (PCA) and visualization using the “FactoMineR” package and “factoextra” package of R software (version 4.0.3). To screen for DEPs and DPPs, we used an independent sample *t*-test. We then applied the Benjamin and Hochberg method to correct the *p*-value for multiple testing and to obtain the false discovery rate (FDR) value. The screening criteria were a fold change greater than 1.2 or less than 0.83, and an FDR less than 0.05. We used Wolfpsort (https://wolfpsort.hgc.jp/) to predict the subcellular structural localization of these proteins. GO and KEGG enrichment analyses were performed using the “clusterProfiler” package of R software (version R-3.5.1). Motif analysis was conducted using soft motif X. We imported the protein list into the STRING (accessed on 12 July 2021) (https://www.string-db.org/) to export the relationship pairs and visualize the protein–protein interaction (PPI) network using Cytoscape software (version 3.8.2). We calculated the degree of each protein in the network using the CytoHubba. A higher degree value indicates a more central position for the protein in the network. We used the MCODE plug-in to filter the core sub-network, with a K-Core set to 2.

### 5.13. Cytotoxicity Assay

In this experiment, we used two inhibitors, SPHINX31 and SRPIN340. MDCK cells were grown in 96-well plates at a density of 5 × 10^4^ cells/mL for 16 h. The cells were then washed two or three times with PBS, and different concentrations of SPHINX31 and SRPIN340 (ranging from 2.5 to 160 μM, diluted by double volume) were added to the wells. The control group received only medium, and three replicate wells were set for each concentration. The cells were incubated at 37 °C for 12, 24, 36, or 48 h. After the incubation period, 5.37 nM GST-ETX was added to the corresponding well and incubated in the cell incubator for 1 h. To estimate cell survival, 100 μL DMEM complete medium and 20 μL MTS (3-(4,5-dimethylthiazol-2-yl)-5(3-carboxymethoxyphenyl)-2-(4-sulfophenyl)-2H-tetrazolium inner salt) were added to the pore and absorbance at 492 nm was measured. As a positive control, cells were treated with toxin only (no inhibitor), and as a negative control, cells were treated with DMEM without the toxin and inhibitor.

### 5.14. Lentivirus-Mediated RNA Interference Assay

Six RNAi constructs were used to specifically knock down the expression levels of NOP56, SF3B1, SF3B2, THOC2, SRSF1, and SRPK1 proteins using lentivirus. MDCK cells were seeded in 96-well plates at 5 × 10^3^ cells per well and cultured for 16 h. The cells were then gently washed two or three times with PBS. The lentivirus was diluted to a working concentration of 5 × 10^7^ TU/mL (MOI = 100), and the lentiviral infection system (86 μL DMEM, 10 μL lentivirus, 4 μL 25 × HiTransG P) was added to the cells which were then incubated in the cell incubator for approximately 16 h. The culture was continued with DMEM culture medium and infected for about 48 h. After that, the cells were treated with different concentrations of diluted GST-ETX (0, 1.79, 3.58, 5.37, 7.16, 8.95 nM) with three replicate wells for each concentration, and incubated in the cell incubator for 1 h. Cell survival was estimated by adding 100 μL DMEM complete medium and 20 μL MTS to the wells and measuring absorbance at 492 nm. Lentivirus and toxin were not added as negative controls, while only toxin cells were added as positive control.

Western blotting was performed to determine if the lentivirus reduced protein levels. In simple terms, after infecting MDCK cells with lentivirus, total protein was extracted using RIPA lysate, separated by 4–20% SDS-PAGE at 130 V for 1 h, and transferred to a PVDF membrane. The membrane was then gently blocked with 5% skim milk at room temperature for 3 h, probed with SRPK1 rabbit antibody (1:1000, Immunoway YT4422, Plano, TX, USA), incubated overnight at 4 °C, and washed three times using PBST. The membranes were incubated with goat anti-rabbit IgG secondary antibody for 1 h. Finally, the color development solution was added, and the results were analyzed and imaged using an AE-1000 cool CCD image analyzer.

### 5.15. RT-qRCR

The knockdown efficiency of lentivirus was verified by RT-qPCR. After MDCK cells were infected with lentivirus, the cells were collected. Total RNA in cells was extracted by Trizol and then quantified using Qubit 4 Fluorometer (Invitrogen, Carlsbad, CA, USA). After treatment with *EVO M-MLV* One Step RT-qPCR Kit (SYBR) (Accurate Biotechnology, Code NO. AG11732, Hunan, China), the detection was carried out on the QuantStudio^TM^ 7 Pro Real-Time PCR System (Thermo Fisher Scientific, Waltham, MA, USA). Relative gene expression was calculated by the 2^−ΔΔCt^ method [48]. Primer information is shown in Table S8.

### 5.16. Statistical Analysis

Statistical analysis was performed with GraphPadPrism9.0 software. The data were presented as the mean ± standard deviation (SD) of multiple independent experiments. Cell survival was logit transformed, and then the differences between groups were analyzed by independent sample one-way ANOVA. We prescribed that the significance level was *p* < 0.05, and the difference was considered to be statistically significant.

## Figures and Tables

**Table 1:** Common differentially expressed proteins in 10 min group and 30 min group.

Protein Accession	Protein Description	Gene me	FC10	FC30	FDR10	FDR30	Type
E2RED7	Bromodomain adjacent to zinc finger domain 1B	BAZ1B	1.328	1.33	0.009233	0.01666	up
J9PAY8	DNA topoisomerase 2	TOP2A	1.295	1.257	0.009233	0.013718	up
E2R5T9	Actinin alpha 4	ACTN4	0.727	0.665	0.011489	0.015039	down
F1PLS4	Vimentin	VIM	0.697	0.595	0.011489	0.016655	down
E2RNB0	Histone H2A	H2AX	0.505	0.471	0.014266	0.004933	down
L7N0L3	Histone H4	H4C7	0.699	0.598	0.015655	0.004933	down
E2RR73	Histone cluster 1 H1 family member a	HIST1H1A	0.647	0.492	0.016482	0.005695	down
E2QY07	Actinin alpha 1	ACTN1	0.831	0.727	0.016911	0.004933	down
F1Q0N9	Keratin 19	KRT19	0.753	0.699	0.025294	0.005341	down
F1Q272	WAS/WASL interacting protein family member 2	WIPF2	0.633	0.665	0.025294	0.028942	down
F1PB61	TATA-box binding protein associated factor 15	TAF15	0.812	0.713	0.025522	0.029035	down
F1PRB0	IF rod domain- containing protein	KRT86	0.696	0.622	0.025522	0.010853	down
F1PW86	Inositol 1,4,5-trisphosphate receptor type 1	ITPR1	0.778	0.721	0.026721	0.033783	down
E2REU6	F rod domain- containing protein	KRT18	0.751	0.669	0.028972	0.015039	down
E2RJ54	Heterogeneous nuclear ribonucleoprotein K	HNRNPK	0.727	0.792	0.029708	0.029035	down
L7N071	Actinin alpha 4	ACTN4	0.777	0.732	0.029708	0.028942	down
E2R6	PEST proteolytic sigl containing nuclear protein	PCNP	0.703	0.612	0.034266	0.007628	down

Protein accession: protein accession number of database used for search, Canis lupus familiaris (Dog) database (ID: 9615). Protein description: protein functional description. Gene me: indicates the name of the gene that code for the protein sequence. FC10: fold change of 10 min. FC30: fold change of 30 min. FDR10: false discovery rate of 10 min. FDR30: false discovery rate of 30 min.

**Table 2:** Common Top5 up-regulated and Top5 down-regulated protein phosphorylation site in 10 min group and 30 min group.

Protein Accession	Protein Description	Gene me	Phosphosites	FC10	FC30	FDR10	FDR30	Type
F1PLN6	Tripartite motif containing 33	TRIM33	S987	0.181	0.104	0.006239	0.03292	down
F1PU14	PDGFA associated protein 1	PDAP1	Y71	0.202	0.252	0.00203	0.002436	down
F6XZH6	Ladinin 1	LAD1	S462	0.503	0.271	0.014744	0.001249	down
F1Q2Z4	Serine/arginine repetitive matrix 2	SRRM2	S2394	0.728	0.327	0.017377	0.003296	down
F1PKT2	Lipolysis stimulated lipoprotein receptor	LSR	S592	0.437	0.333	0.005074	0.001034	down
J9P969	AHK nucleoprotein	AHK	S4691	3.607	3.563	0.003419	0.007272	up
E2RR55	Nuclear factor of activated T cells 2 interacting protein	NFATC2IP	S90	2.512	3.230	0.03395	0.000727	up
J9NRJ1	40S ribosomal protein S6	---	S235	2.604	3.169	0.001298	0.001109	up
E2RJ15	Myotubularin related protein 10	MTMR10	S600	4.211	3.095	0.001298	0.003654	up
J9NRJ1	40S ribosomal protein S6	---	S236	2.278	3.077	0.00203	0.000994	up

Protein accession: protein accession number of database used for search, Canis lupus familiaris (Dog) database (ID: 9615). Protein.description: protein functional description. Gene me: indicates the name of the gene that code for the protein sequence. FC10: fold change of 10 min. FC30: fold change of 30 min. FDR10: false discovery rate of 10 min. FDR30: false discovery rate of 30 min. ---: the gene name is not currently available in the database.

**Table 3:** Analysis and arrangement of KEGG pathways of 13 homologous key proteins in the cluster in 10 min group and 30 min group.

Gene me	Protein Accession	Type10	Type30	Subcellular Localization	KEGG Pathway
SRSF1	E2RJL3	down	down	nucleus	cfa04657; cfa05168; cfa03040
SRSF2	E2RDB2	up	up	nucleus	cfa05168; cfa03040
SRSF6	F1PTE0	up	up	nucleus	cfa05168; cfa03040
SRSF7	J9P2I8	down	up	nucleus	cfa05168; cfa03040
SF3B1	F1PKF6	up	up	plasma membrane	cfa03040
SF3B2	E2RL65	up	up	nucleus	cfa03040
GTPBP4	F1PJY9	up	up	nucleus	cfa03008
THOC2	J9PB31	down	down	cytoplasm	cfa03013; cfa03040
NOP56	E2QU53	up	up	nucleus	cfa03008
NOP14	F1PI93	up	up	plasma membrane	0
ANLN	F1PZ90	up	up	nucleus	0
SRSF11	F1P6U8	down	down	nucleus	0
RRP1B	F1PS87	down	down	cytoplasm, nucleus	0

Protein accession: protein accession number of database used for search. Gene me: indicates the name of the gene that code for the protein sequence. KEGG: pathways of proteins in KEGG database.

## Data Availability

The datasets generated during and/or analyzed during the current study are available from the corresponding author upon reasonable request.

## Supplementary Materials

The following supporting information can be downloaded at: https://www.mdpi.com/article/10.3390/toxins16090394/s1, Figure S1: Subcellular localization statistics; Table S1: Differential protein information; Table S2: Phosphorylation sites information; Table S3: All up- and down-regulated DPPs and DEPs’ information; Table S4: All DPPs’ information; Table S5: Motif-X analysis of significantly regulated phosphopeptides; Table S6: Cytohubba results Top 20 degree. Table S7: RNAi-customized-lentivirus information; Table S8: Primer information for RT-qPCR.
