# Supplementary material for: Proteome and Phosphoproteome Profiling Reveal the Toxic Mechanism of Clostridium perfringens Epsilon Toxin in MDCK Cells
Source: Toxins (Basel). 2024 Sep 14;16(9):394. doi: 10.3390/toxins16090394 (PMC11435651; doi:10.3390/toxins16090394)
Supplement: Supplementary file 1 [file toxins-16-00394-s001.zip › Figure S1.pdf]

Support information

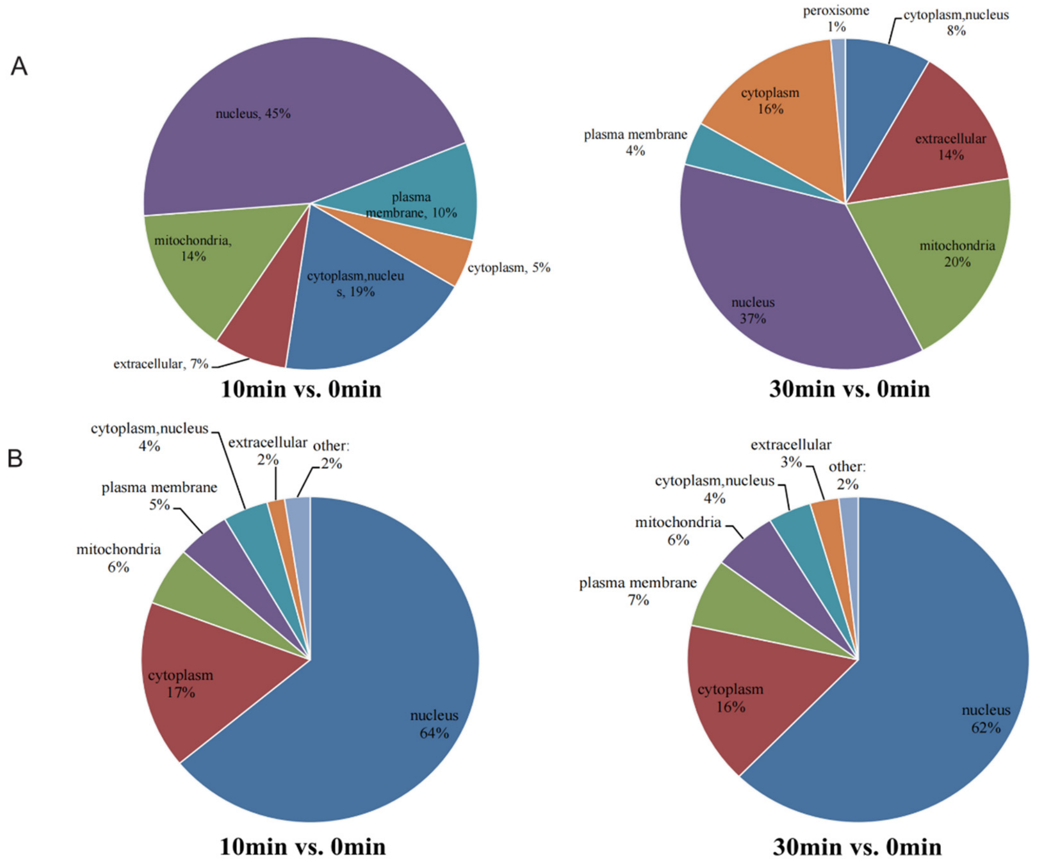

Figure S1. Subcellular localization statistics. (A) Subcellular localization of differentially expressed proteins. (B) Subcellular localization of differentially phosphorylated proteins.
