# Supplementary material for: Proteome and Phosphoproteome Profiling Reveal the Toxic Mechanism of Clostridium perfringens Epsilon Toxin in MDCK Cells
Source: Toxins (Basel). 2024 Sep 14;16(9):394. doi: 10.3390/toxins16090394 (PMC11435651; doi:10.3390/toxins16090394)
Supplement: Supplementary file 1 [file toxins-16-00394-s001.zip › Table S7.pdf]

Table S7 RNAi-customized-lentivirus information

Gene name	Gene ID	Gene bank	KEGG pathway	CDS Size	Lentiviral vector
SRPK1	474887	XM_038683302.1	cfa05168	2484	GV493
SRSF1	609693	XM_038677056.1	cfa05168/cfa04657	747	GV493
NOP56	612871	XM_038433312.1	cfa03008	1794	GV493
SF3B1	488456	XM_O38447486.1	cfa03040	3915	GV493
SF3B2	476015	XM_038424919.1	cfa05168/cfa03040	2688	GV493
THOC2	481042	XM_038450799.1	cfa03040	4875	GV493
