# Supplementary material for: Proteome and Phosphoproteome Profiling Reveal the Toxic Mechanism of Clostridium perfringens Epsilon Toxin in MDCK Cells
Source: Toxins (Basel). 2024 Sep 14;16(9):394. doi: 10.3390/toxins16090394 (PMC11435651; doi:10.3390/toxins16090394)
Supplement: Supplementary file 1 [file toxins-16-00394-s001.zip › Table S8.pdf]

Table S8 Primer information for RT-qPCR

Gene name	Sequence (5'to3')
β-actin-F	GCTTCTTTGCAGCTCCTTCG
β-actin-R	CCTTCTGACCCATTCCCACC
SRPK1-F	GAATGAGCAGTACATCCGAAGAC
SRPK1-R	CTGACCGCAGATCCAGAAGG
SRSF1-F	ACTGCCTACATCCGGGTTAAAG
SRSF1-R	CGTGGTGATCCTCTGCTTCTC
NOP56-F	CAAGGAGGTGGAGGAGATCAG
NOP56-R	CAAGAGCAGGCGGAGGTC
SF3B1-F	GCACGCGCAGAGAGCATTC
SF3B1-R	CACTCGAACACACAGACGGAAC
SF3B2-F	CATCCCGAGCCTCCCAAAG
SF3B2-R	CTGTCTGGCGCGTGTAGG
THOC2-F	GCGGTGCTCCCCTTTGAC
THOC2-R	CCTGATTTCTCCCAGTTCTTTATCC
